# Circadian clock gene expression and polymorphism in non-segmental vitiligo

**DOI:** 10.1007/s11033-023-09109-6

**Published:** 2024-01-18

**Authors:** Azza Gaber Antar Farag, Eman A. E. Badr, Asmaa Fahmy Ibrahim

**Affiliations:** 1 Andrology and S.T.Ds, Faculty of Medicine, Menoufia University, Shebin AlKom, Egypt; 2 Medical Biochemistry and Molecular Biology Department, Faculty of Medicine, Menoufia University, Shebin AlKom, Egypt; 3 Department of Clinical and Molecular Parasitology, National Liver Institute, Menoufia University, Shebin AlKom, Egypt

**Keywords:** Non-segmental vitiligo, Circadian gene, BMAL1 gene, Lipid profile

## Abstract

**Background:**

Vitiligo is an acquired and progressive mucocutaneous disease with the damage of functioning epidermal melanocytes. Metabolic syndrome is associated with inflammatory skin diseases incorporating vitiligo. The circadian dysfunction triggers the pathogenesis of metabolic diseases, so our study aimed to determine the relationship between aryl hydrocarbon receptor nuclear translocator-like gene, a ligand-activated transcription factor and sensor of environmental chemicals, expression and polymorphism with non-segmental vitiligo, as well as its effect on lipid profile.

**Methods:**

This case-control study was handled on 50 non-segmental vitiligo patients (generalized (12) and localized type (focal; 24 and acrofacial; 14)) and 50 matched controls. Each subject was proposed for full history taking, clinical examinations, serum lipid profile, and measurement of BMAL1 gene expression in the blood, and BMAL1 rs2279287 polymorphism of DNA extract from whole blood by real time-PCR.

**Results:**

We identified that total cholesterol, triglyceride, and low-density lipoprotein were significantly higher, but high-density lipoprotein was significantly lower in non-segmental vitiligo patients than in the control group. A significant increase in circadian gene expression in non-segmental vitiligo patients was observed, with more detection of the BMAL1 T/C genotype (92%) than the T/T genotype. There was a significant positive relationship between the level of the circadian gene and the vitiligo patient’s age, age of onset, and VIDA Score. The level of the circadian gene at Cutoff  ≥ 1.16 can predict the prognosis of vitiligo with a sensitivity of 78%, specificity of 84%, and accuracy of 81%.

**Conclusion:**

The circadian gene has an active role in the progress of non-segmental vitiligo and targeting this gene could have a significant impact on its management.

## Introduction

Vitiligo is an autoimmune pigmentary disorder that damages melanocytes in the skin. It can develop before the age of 20 or at any age, with non-significant differences between sexes [1]. It is described by acquired, idiopathic, progressive, circumscribed hypomelanosis of the skin and hair. It appears worldwide, with an incidence rate of between 0.1% and 2%. Vitiligo is a critical skin disease having a chief effect on the quality of life of the patient enduring it. The causes of this disorder are doubtful but look to be determined by the interface of genetic, immunological and neurological aspects. Since its pathogenesis is yet not recognized, there is an excess of several treatments. Including, topical steroids and narrowband ultraviolet B monotherapy [2].

There are two categories of vitiligo, segmental (SV) and non-segmental (NSV). NSV has genetic factors that increase the risk of autoimmunity [3], and it shows a more prominent modification of the immune response and B cell differentiation than SV as evident by the presence of anti-melanocyte antibodies, increased proportion of circulating memory B cells, and decreased levels of naive B cells [4].

External signals like light and temperature can affect the circadian system in humans, which is controlled by a central pacemaker in the hypothalamus, this system is responsible for various endocrinal, physiological, and behavioral activities such as body temperature, hormone secretion, and the sleep-wake cycle [5].

The molecular circadian clock is composed of positive and negative regulators, which activate and repress transcription via E-box which is a DNA response component that performs as a protein-binding site and adjusts gene expression. It has a specific DNA sequence which is identified and bound by transcription factors to begin gene transcription. When the transcription factors bind to the promoters through the E-box, other enzymes can bind to the promoter and facilitate transcription from DNA to mRNA. The second group of agents that involve Cryptochrome (CRY1, CRY2) and Period (PER1, PER2, PER3) proteins form the first assembly and initiate transcription through E-boxes. CRYs repress upon binding as CRY-CLOCK-BMAL1-E-box complexes, whereas Period proteins (PERs) repress by eliminating the heterotrimeric complexes from the E-box [6]. The auxiliary loop of REV-ERB/ROR keeps the system’s steadiness which is a mechanism that supports in the regulation of molecular clocks and includes a protein called Rev-Erb alpha (α), which oscillates with PER and CRY over a 24 h period, helping in the process of inhibiting CLOCK and BMAL1 heterodimers from forming in the nucleus. It also plays an important role in various physiological activities throughout the body, incorporating metabolic, endocrine, and immune pathways [7]. Every normal cell progresses via these physiological phases in chronological order [8].

BMAL1/ARNTL-1is a transcription factor that is part of the circadian clock protein. It creates circadian rhythms in physiological purposes, involving the inflammatory response of macrophages to manipulate the production of pro-IL-1β. The aryl hydrocarbon receptor (AhR) is extensively expressed in immune cells, crucial for immune responses and its abnormal signaling may be linked to autoimmune diseases. It is stimulated by ligands that present in the environment, implicated in the regulation of glucose and lipid metabolism, and its dysregulation has been correlated to metabolic syndrome. It also plays a fundamental role in the regulation of circadian rhythms [9], so this research aimed to study the connection between the aryl hydrocarbon receptor nuclear translocator-like gene expression and polymorphism with NSV and its effect on the lipid profile.

## Subjects and methods

### Study design

Our study was operated on 50 NSV patients aged 18 years or older who joined the Outpatient Clinic of Dermatology, Faculty of Medicine, Menoufia University between December 2021, and October 2022. A control group of 50 healthy individuals who had no family history of vitiligo and were corresponding for age was also included.

This study was approved by the Ethical Committee of Human Rights in Research at the Faculty of Medicine Menoufia University following the Helsinki Declaration in 1975 (revised in 2000) and has an ethics committee approval number of (DERM 39/2022). A written consent was obtained from the cases and controls or their legal guardians.

**Table:** 

The inclusion criteria	The exclusion criteria
• Non segmental vitiligo	• Segmental or mixed vitiligo
• Male or female aged 18 years or older	• Systemic diseases such as diabetes mellitus, cirrhosis, infection, renal failure, thyroid, and connective tissue diseases
• Type of vitiligo: generalised and localised	• Autoimmune diseases (systemic or cutaneous) such as rheumatoid arthritis, atopic dermatitis, and psoriasis,
• Distribution of vitiligo: focal and acrofacial	• Those who received systemic corticosteroids and/or other immune suppressants throughout the last month, and
• Disease activity using vitiligo disease activity score (VIDA score): 0 up to +4	• Those who received any systemic (within 6 weeks) or topical (within 2 weeks) treatment of vitiligo
• The percentage of body area involved and vitiligo extent using vitiligo extents core (VES) was measured	• Pregnant and lactating patients
• Associated mucosal involvement	• Smokers
• Presence of leukotrichia	• Drug abusers or addictions

### Methods

Each participant underwent a full history taking and clinical examination, including a dermatological examination to confirm the diagnosis of NSV. Disease severity and activity were assessed via the vitiligo area scoring index (VASI) [10] and vitiligo disease activity (VIDA) scoring [11]. Height and weight were determined to estimate body mass index (BMI) [12].

### Blood sampling

Blood samples were collected from every participant with absolute aseptic conditions and after overnight fasting. 5 mL of blood was taken and separated into two parts. One part, 2 mL, was stored in an EDTA tube for RNA&DNA extraction. The other part, 3 mL, was put in a plain tube, set to clot for 30 min, and then went through centrifugation for 10 min at 4,000 × g per minute. The acquired serum was kept until the time for the lipid profile. All laboratory investigations were conducted in the Medical Biochemistry and Molecular Biology Department, Faculty of Medicine, Menoufia University.

### Assay methods

#### Lipid profile

A colorimetric enzymatic method was used to quantitatively estimate total cholesterol (TC), high-density lipoprotein (HDL)-cholesterol, and triglycerides (TG). Standard enzymatic colorimetric kits from Spinreact Diagnostics Kit, Spain, were used for this purpose. Low-density lipoprotein (LDL)-cholesterol was executed by the modified Friedewald equation [13].

#### RNA isolation

RNA was isolated from the blood using a Direct-zol RNA Miniprep kit from Zymo Research. A two-step RT-PCR was accomplished, with the first step being cDNA synthesis (RT-Step). The Quanti-Tect Reverse Transcription Kit from Qiagen, Applied Biosystems, USA, 2012 was handled for the reverse transcription step. Samples were all set in a volume of 20 µL including 4 µL RT buffer, 1 µL reverse transcriptase (Applied Biosystems), and 10 µL of extracted RNA and 5 µL of DNase/RNase-free water. The samples were afterward incubated at 25 °C for 10 min and at 48 °C for 30 min on the 2720 thermal cycler Singapore. Heating up to 95 °C for 5 min inhibited the reverse transcriptase. The generated cDNA was stored at 20 °C. The subsequent step of PCR was cDNA amplification with SYBR Green II with low ROX for revealing Bmal1 gene expression (Quanti-Tect SYBR Green PCR Kit, Applied Biosystems, USA) [14].

#### Quantitative real-time PCR (qRT-PCR)

**Fig. 1:**
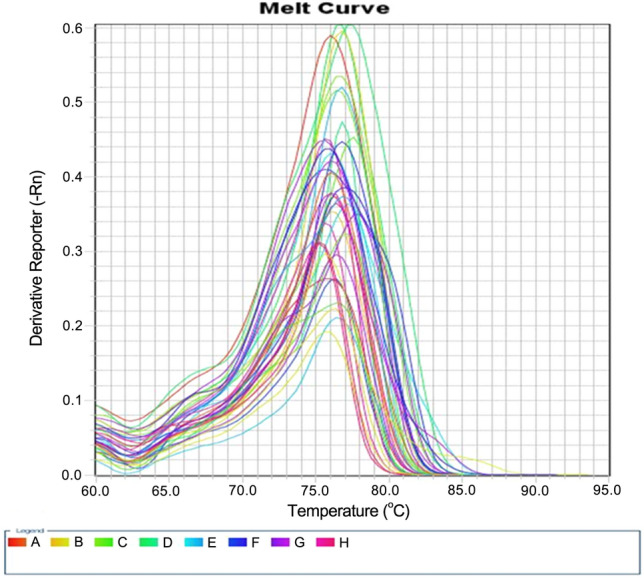
The melting curve

The cDNA was employed in a SYBR green-based qRT-PCR that was operated with the Quanti-Tect SYBR Green PCR Kit with a convenient Quanti-Tect Primer Assay (Qiagen). The reaction was carried out by mixing 12.5 µL 2x Quanti-Tect SYBR Green PCR Master Mix, 5 µL cDNA, 1 µL of every primer, and 5.5 µL RNase-free water. The reaction progressed as 45 cycles; 30 s at 94 °C for denaturation; 30 s at 55 °C for annealing; and 30 s at 72 °C for the extension. The reaction mix was assembled for each primer in a separate well, with a total reaction volume of 20 µL final for each [15]. The melting curve is shown in Fig. 1 and the amplification plot displaying the level of the BMAL1 gene expression is shown in Fig. 3a and b.

#### Genotyping assay

DNA was purified with a Qiagen DNA extraction kit (Hilden, Germany) with the company’s procedure protocol. Allelic discrimination assays using TaqMan probes were utilized to genotype BMAL1 rs2279287 (Applied Biosystems, USA). 5 µL of sample DNA was added to a combination of 10 µL of genotyping master mix, 1.25 µL of primer/probe assay, and 3.75 µL of DNAase-free water to produce an entire mix of 20 µL. The TaqMan probes were labeled with VIC and FAM fluorescent dyes. Cycling provisions were accomplished at an initial 50 °C for 60 s, then 95 °C for 10 min as a primary denaturation step followed by 45 cycles of 15 s at 95 °C and 60 s at 60 °C (cycling), and a last extension step for 60 s at 60 °C [16]. The allelic discrimination plot of the SNP of the BMAL1 rs2279287 gene is shown in Fig. 3b.

**Table:** 

The primers	5′-TGCAAGGGAAGCTCACAGTC-3′ (forward)
	5′-GATTGGTGGCACCTC TTAATG-3′ (reverse)
GAPDH	CCACTC CTC CACCTTTGAC (forward)
	ACCCTGTTGCTGTAGCCA (reverse)
The probe sequence	GCGCTGGCGCGGGGCTGTGTCTAC [C/T]
	CTTCAGTAAGTGGTCAAAACCTG [GC]

#### Statistical analysis

We used the SPSS (Statistical Package for Social Science) program version 20. Quantitative data were presented as mean, standard deviation (SD), and median (25th, 75th), while qualitative data were stated as frequency and percentage. The chi-square test or Fisher exact test was handled to measure the association amongst qualitative variables, and the Mann–Whitney test, or independent sample *t* test was used to detect the significance between two quantitative variables. The correlation between two continuous variables was measured using Spearman correlation analysis. The level of the circadian gene was evaluated using the receiver operating characteristic (ROC) curves. A probability (p)-value less than 0.05 was deemed statistically significant.

## Results

### Baseline characteristics and clinical presentations

**Fig. 2:**
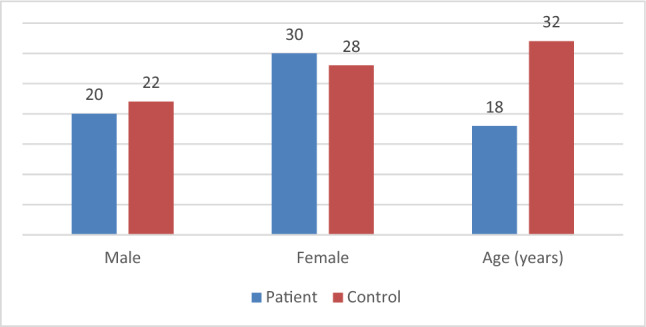
The age and gender both are comparable between vitiligo patients and controls

In this study, it was found that the median age of vitiligo patients was 18 years, which is comparable to that of the control group (32 years). Females were more affected in this study, with 60% of patients being female. Nearly half of the patients had a gradual onset (new patches gradually develop over a year) and stationary course (the depigmentation remains stable over 2 years) of the disease. Family history was present in 16% of patients, while leukotrichia was present in 14%. The median disease duration was 24 months, and no spontaneous regimentation (the natural re-pigmentation of the skin in areas affected by vitiligo without any medical intervention) was detected. Previous treatment was received by 96% of vitiligo patients, and 78% of patients showed improvement after treatment. The median of the VIDA and VASI scores were 2 and 3.5, respectively as shown in Table 1; Fig. 2.

**Table 1:** General characteristics of the studied groups

Patient group	Patient (N = 50) N (%)		
Gender	Male		20 (40%)
	Female		30 (60%)
Age (years)	Mean ± SD		23.5 ± 14.49
	Median (25th–75th)		18 (11–32.25)
Age of onset (years)	Mean ± SD		20.88 ± 13.184
	Median (25th–75th)		18 (10–28.5)
Onset	Sudden		22 (44.0%)
	Gradual		28 (56.0%)
Course	Stationary		26 (52.0%)
	Progressive		20 (40.0%)
	Regressive		4 (8.0%)
Duration (months)	Mean ± SD		32.24 ± 34.103
	Median (25th–75th )		24 (12–48)
Family history	Absent		42 (84.0%)
	Present		8 (16.0%)
The extent of the involvement	Generalized		12 (24%)
	localized	Focal	24 (48%)
		Acrofacial	14 (28%)
Previous treatment	Yes		48 (96.0%)
	No		2 (4.0%)
Response to treatment	Improved		39 (78.0%)
	Not improved		11 (22.0%)
Spontaneous repigmentation	No		50 (100.0%)
Leukotrichia	Yes		7 (14.0%)
	No		43 (86.0%)
VIDA score	Mean ± SD		1.96 ± 1.10
	Median (25th–75th)		2 (1–3)
VASI score	Mean ± SD		3.61 ± 1.104
	Median (25th–75th)		3.5 (2.5–4.55)

Z (Mann–Whitney test), t (independent sample *t* test)

### Lipid profile and BMI

We detected that TC, TG, and LDL were significantly higher, but HDL was significantly lower in non-segmental vitiligo patient than in the control group (P < 0.0001). while the BMI was comparable between the 2 groups (P > 0.05) as the BMI median was 32 Kg/m^2^ in vitiligo patients which was more than that of the control group (29 Kg/m^2^) (Table 2).

**Table 2:** Comparison of the lipid profile and BMI between patient and control groups

Lipid profile	Patient N = 50 Mean ± SD Median (25th–75th)	Control N = 50 Mean ± SD Median (25th–75th)	Mann–Whitney test	P-value
Total cholesterol (mg/dL)	205.1 ± 47.6	155 ± 16.5	t = 5.35	< 0.0001
	208 (181–243)	155.5 (145–166)		
Triglyceride (mg/dL)	148 ± 51.4	111 ± 17.4	4.44	< 0.0001
	140 (111.5–183.5)	105 (100–120)		
Low-density lipoprotein (mg/dL)	124.8 ± 45.9	86 ± 17.84	5.61	< 0.0001
	110.5 (99.75–170)	85 (78–96)		
High-density lipoprotein (mg/dL)	39.98 ± 6.76	47.84 ± 3.41	t = 7.43	< 0.0001
	39 (37–42)	48 (45–50)		
Body mass index (Kg/m^2^)	30.84 ± 4.38	29.3 ± 3.68	t = 1.55	0.119
	32 (26.75–34)	29 (26–33.25)		

t (independent sample *t* test)

### BMAL1 expression and polymorphism

**Fig. 3:**
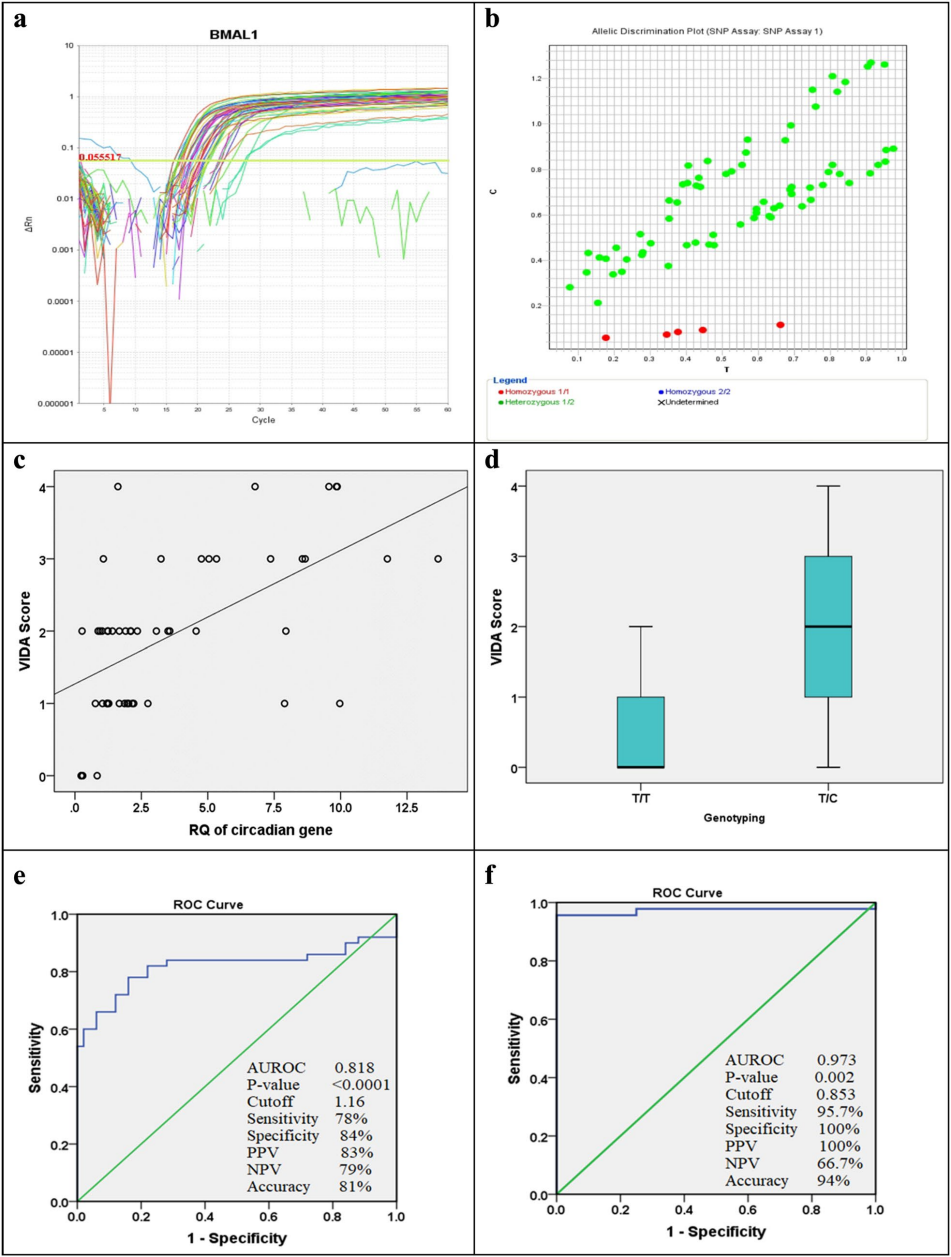
Amplification plot showing the expression level of the BMAL1 gene in vitiligo patients (**a**). Allelic discrimination plot of SNP of BMAL1 rs2279287 gene in vitiligo patients (**b**). Scatter dot showing a correlation between the level of the circadian gene with VIDA score in the vitiligo patient group, with each point in the figure resembling the VIDA score value for a single patient (**c**). Box and whiskers plot showing VIDA in the vitiligo patient with T/T and T/C genotypes. The upper and lower transverse lines of the box show the 75th and 25th percentile correspondingly and the line in between shows the median with the whiskers’ upper and lower transverse lines showing the maximum and minimum levels respectively (**d**). ROC curve showing that the level of the circadian gene at Cutoff  ≥ 1.16 could diagnose vitiligo with a sensitivity of 78%, specificity of 84%, and accuracy of 81% (**e**). The ROC curve shows that the level of the circadian gene at Cutoff  ≥ 0.853 could suspect TC genotype with a sensitivity of 95.7%, specificity of 100%, and accuracy of 94% (**f**)

Our study demonstrated a significant (< 0.0001) increase in circadian gene expression in NSV patients than in the control group, with more detection of the BMAL1 T/C genotype (92%) (Table 3). A significant positive relation was noticed between the level of the circadian gene and the vitiligo patient’s age, age of onset, and VIDA Score (P < 0.05), but comparable with VASI, the site of involvement, lipid profile and BMI (Table 4; Fig. 3c).

The present study presented no statistically significant difference between vitiligo patients with T/C and T/T genotypes regarding the clinical presentation (P > 0.05) except VIDA score which was statistically significantly higher in patients with the T/C genotype (< 0.0001). The T/C genotype was more detected in females (60.9%) but the T/T genotype was equal in both genders. As regards T/C genotype, gradual onset of 56.5%, stationary course of 52.2%, absent family history of 87.0%, and absent leukotrichia of 84.8% were detected with a median age of onset of 18 years and median duration of 24 months in comparison to T/T genotype which were comparable. However, regarding T/T genotype, no leukotrichia was detected, with a median age of onset of 12.5 years, and a median duration of 36 months. The level of the circadian gene was significantly higher in vitiligo patients with the T/C genotype (P = 0.002) (Table 5; Fig. 3d).

**Table 3:** Circadian clock gene expression and polymorphism in vitiligo patients and control groups

Circadian clock gene expression and polymorphism		Patient, N = 50 N (%)	Control, N = 50 N (%)	X2	P-value
Genotyping	T/T	4 (8%)	16 (32%)	9	0.003
	T/C	46 (92%)	34 (68%)		
RQ of circadian gene	Mean ± SD	3.72 ± 3.50	1.036 ± 0.286	Z = 5.51	< 0.0001
	Median (25th–75th)	2.097 (1.21–5.69)	1 (0.87–1.034)		

Z (Mann-Whitney test)

**Table 4:** Circadian gene level and the vitiligo patients’ parameters

Patient group	RQ of circadian gene	
	r	P
Age	0.309	0.029
Duration	0.059	0.683
Age of onset	0.314	0.027
VIDA Score	0.587	< 0.0001
VASI Score	0.068	0.641
Total cholesterol	− 0.046	0.752
Triglyceride	0.135	0.350
Low-density lipoprotein	− 0.0128	0.376
High-density lipoprotein	0.269	0.059
Body mass index	0.068	0.638
The site of involvement	0.090	0.554

**Table 5:** The clinical presentation in vitiligo patients with T/T and T/C genotypes

Patient group	Genotyping			Fisher exact test	P-value
		T/T N = 4	T/C N = 46		
Gender	Male	2 (50.0%)	18 (39.1%)	0.181	0.670
	Female	2 (50.0%)	28 (60.9%)		
Age (years)	Mean ± SD	14.75 ± 4.99	24.26 ± 14.8	Z = 1.077	0.281
	Median (25th–75th)	14.50 (10.25–19.50)	18 (11.75–33.25)		
BMI (Kg/m^2^)	Mean ± SD	31.75 ± 6.13	30.76 ± 4.28	t = 0.054	0.957
	Median (25th–75th )	32 (26–37.25)	32 (26.75–34)		
Onset	Sudden	2(50.0%)	20(43.5%)	0.064	0.801
	Gradual	2(50.0%)	26(56.5%)		
Course	Stationary	2(50.0%)	24(52.2%)	0.460	0.795
	Progressive	2(50.0%)	18(39.1%)		
	Regressive	0(0.0%)	4(8.7%)		
Family history	Absent	2(50.0%)	40(87.0%)	3.740	0.053
	Present	2(50.0%)	6(13.0%)		
Leukotrichia	Yes	0(0.0%)	7(15.2%)	0.708	0.400
	No	4(100.0%)	39(84.8%)		
Duration	Mean ± SD	32.50 ± 18.78	32.22 ± 35.25	Z = 0.398	0.691
	Median (25th–75th )	36 (13.5–48)	24 (12–48)		
Age of onset	Mean ± SD	12.25 ± 6.65	21.63 ± 13.3	Z = 1.469	0.142
	Median (25th–75th )	12.5 (6.25–18)	18 (10–30.25)		
Previous treatment	Yes	4 (100%)	44 (95.7%)	0.181	0.670
	No	0 (0%)	2 (4.3%)		
Response to treatment	Improved	3 (75.0%)	36 (78.3%)	0.023	0.880
	Not improved	1 (25.0%)	10 (21.7%)		
VIDA score	Mean ± SD	0.50 ± 1	2.09 ± 1.029	Z = 2.504	0.012
	Median (25th–75th )	0 (0–1)	2 (1–3)		
VASI score	Mean ± SD	3.38 ± 0.64	3.63 ± 1.13	t = 0.197	0.844
	Median (25th–75th )	3.3 (2.85–4)	3.5 (2.5–4.55)		
RQ of circadian gene	Mean ± SD	0.41 ± 0.284	4.018 ± 3.506	3.111	0.002
	Median (25th–75th )	0.279 (0.25–0.7)	2.185 (1.26–6.92)		

Z (Mann–Whitney test), t (independent sample *t* test)

### ROC curves

The level of the circadian gene at Cutoff ≥ 1.16 can predict the prognosis of vitiligo with a sensitivity of 78%, specificity of 84%, and accuracy of 81% (Fig. 3e). Furthermore, the level of the circadian gene at Cutoff ≥ 0.853 can suspect TC genotype with a sensitivity of 95.7%, specificity of 100%, and accuracy of 94% (Fig. 3f).

## Discussion

Metabolic syndrome (MetS) is a medical disorder identified when a person has three or more of the following traits: large waist, reduced HDL cholesterol, increased blood pressure and elevated fasting blood sugar. It can eventually lead to cardiovascular complications [17], affecting approximately 25% of the global residents. This condition is associated with many inflammatory skin diseases, including vitiligo [18].

Research in molecular epidemiology has shown that discrepancies in circadian rhythm can increase the progression of various diseases [19]. The incidence of several cancers [20, 21], metabolic [22], and mood disorders [23] has been related to polymorphic variations in circadian genes.

Circadian genes play a crucial role in regulating gene expression, involving cell proliferation, apoptosis, DNA repair, and cell cycle control. Single-nucleotide polymorphism (SNPs) in these genes can potentially affect a patient’s therapeutic response and survival [24]. The BMAL1 gene, also identified as brain and muscle ARNT-like protein 1 (BMAL1/ARNTL-1), is located on chromosome 11p15.3 and creates heterodimers with CLOCK and NPAS2 (CLOCK-BMAL1, NPAS2-CLOCK) [25].

Since vitiligo is an inflammatory disease, the present research has proposed that the circadian gene might have a function in the interface between inflammatory and metabolic pathways. This research aimed to estimate the correlation between aryl hydrocarbon receptor nuclear translocator-like gene expression and polymorphism with NSV and its relation to lipid profile.

Our research found that the age of vitiligo patients was lower compared to the control group, but there was no significant difference in the age between patients with T/C and T/T genotypes. These results align with previous studies by El-Husseiny et al. indicating that childhood vitiligo varies from adult-onset vitiligo concerning various aspects as early progressive onset of NSV, and several activities leading to koebnerization at various locations [26]. Moreover, It is probable that more alertness by parents could lead to earlier discovery of vitiligo in children, which could clarify why children had a significantly lower age of onset of vitiligo than adults [27]. Another research stated that both forms of vitiligo (SV and NSV) have a comparable early onset [28].

Our study also found that gender was comparable between vitiligo patients and controls and also there was no significant difference in gender between patients with T/C and T/T genotypes. More studies have suggested that more females than males are affected by vitiligo [26, 29], possibly because it is considered a cosmetic problem [30]. It is important to note that women and girls tend to seek consultation more often for non-segmental vitiligo due to a greater negative social impact [31]. However, conflicting research has shown that vitiligo is more frequent in males [32] or has an almost equivalent occurrence in boys and girls [33].

Regarding family history, our study found that 16.0% of patients had a positive history, with no significant difference between patients with T/C and T/T genotypes. Contrary to our results, El-Husseiny et al. stated a greater percentage of children with a positive family history, possibly due to parents being more concerned and bringing their children to the clinic promptly [26]. Some studies suggest that vitiligo has a genetic component, but the genetic risk is not certain [34, 35].

In our study, we found that 14.0% of vitiligo patients had leukotrichia, with no spontaneous regimentation in all studied vitiligo patients. There was no statistically significant difference between patients with T/C and T/T genotypes regarding leukotrichia and spontaneous regimentation. Other studies have reported that leukotrichia is present in nearly third of the studied sample in both children and adults [26, 29]. Others stated that its presence may indicate a lower melanocyte reservoir and a weaker response to treatment [36, 37].

Vitiligo extent score (VES) showed the highest score in adults followed by adolescents then children displaying a statistically significant difference which imply advancement of disease with age. Furthermore, correlation between VES and duration of vitiligo among patients was highly significant as the more duration of vitiligo, the highest VES documented. Additionally, there was a significant association between the VES and time interval of vitiligo, with a higher VES in patients with longer disease duration [26]. Median VIDA and VASI scores in our study were 2 and 3.5, respectively. Patients with the T/C genotype had a significantly greater VIDA score, while there was no significant difference in VASI score between the T/T and T/C genotypes.

Our study also found that vitiligo patients had higher TC, TG, and LDL levels but lower HDL levels compared to the control group. This is agreed with earlier studies that have proven an association between dyslipidemia and vitiligo [38–40]. Cho et al. showed that REV-ERB_ alpha, one of two Rev-Erb proteins in the nuclear receptor family of intracellular transcription factors which shows an important role in regulation of the core circadian clock by repression of the positive clock element Bmal, was associated with high triglyceride levels [41], and others [42] discovered that the danger of developing MetS is augmented in patients with NSV.

However, there are conflicting results regarding HDL and TG levels in different studies [43, 44], possibly due to genetic and racial susceptibility, age groups, and additional risk considerations such as smoking, alcohol drinking, diet, and physical activity [45]. Therefore, individual risks should be considered in the management of vitiligo [46].

In our study, we found that the BMI of vitiligo patients was comparable to that of the control group and there was no statistically significant difference between patients with T/C and T/T genotypes in terms of BMI. These results are consistent with previous studies which also reported no significant difference in weight and height between vitiligo patients and controls but found that patients with vitiligo had a greater BMI [45, 47].

Several studies have endorsed that there is a correlation between the circadian system and inflammatory diseases [48]. Yet, the function of clock genes in the mechanism of inflammation is not entirely known [49]. We reported a significant circadian gene expression in vitiligo patients compared to control, with more detection rate of the BMAL1 T/C genotype. Additionally, a significant positive correlation was monitored between the level of the circadian gene and the patient’s age, age of onset, and VIDA Score. The circadian gene at cutoff ≥ 1.16 could predict the prognosis of vitiligo with a sensitivity of 78%, specificity of 84%, and accuracy of 81%. Also, the circadian gene at Cutoff ≥ 0.853 could suspect TC genotype with a sensitivity of 95.7%, specificity of 100%, and accuracy of 94%.

In a similar study guided by Kaneshiroa et al. on rheumatoid arthritis patients, the diagnostic potential of Per2 with a cutoff of 4.457 showed 80.0% sensitivity and 73.3% specificity. Cry1 with a cutoff of 1.439 displayed 53.3% sensitivity and 73.3% specificity, while Cry2 with a cutoff of 6.989 showed 73.3% sensitivity and 73.3% specificity. Additionally, Clock presented 60.0% sensitivity and 93.3% specificity with a cutoff of 1.461, and Rora with a cutoff of 15.35 confirmed 73.3% sensitivity and 73.3% specificity [50].

The aim of treating vitiligo is to maximize the effectiveness of the treatment while minimizing any negative effects. Recent research has emphasized the importance of considering the individual’s natural rhythm and the bioavailability of the treatment. This means that medications may be tailored to an individual’s inner clock to optimize their effectiveness. Inflammatory diseases can benefit from this approach, as shown in recent studies [51]. In the future, therapies that target the circadian clock may prove effective in managing not only vitiligo but also the accompanying inflammation [49].

## Conclusions

The circadian gene may have a significant utility in the development of non-segmental vitiligo and might be a potential target for treatment. Dyslipidemia may be associated with this gene’s role in the disease process. The circadian gene could be a useful marker for early diagnosis, but it does not help determine the severity of vitiligo. The possible limitation in this study was the low total number of studied subjects.

## Data Availability

The data underlying this article will be shared on reasonable request to the corresponding author.

## Abbreviations

BMI: Body mass index
CRY: Cryptochrome
HDL: High-density lipoprotein
LDL: Low-density lipoprotein
MetS: Metabolic syndrome
NSV: Non-segmental vitiligo
PER: Period
p-value: Probability value
qRT-PCR: Quantitative real-time polymerase chain reaction
ROC: Receiver operating characteristic
SD: Standard deviation
SNPs: Single-nucleotide polymorphism
SPSS: Statistical package for social science
SV: Segmental vitiligo
TC: Total cholesterol
TG: Triglycerides
VASI: Vitiligo area scoring index
VES: Vitiligo extent score
VIDA: Vitiligo disease activity scoring
